# Gross Bimodal Diurnality in Dementia Behavioural Symptoms in an Inpatient Setting: High Noon and Sundown

**DOI:** 10.1192/bjo.2022.167

**Published:** 2022-06-20

**Authors:** David Anderson, Neveen Hamza, Keith Reid, Jonathan Richardson

**Affiliations:** 1 Cumbria, Northumberland, Tyne and Wear NHS Foundation Trust, Newcastle, United Kingdom.; 2 Newcastle University, Newcastle, United Kingdom

## Abstract

**Aims:**

Our purpose-built dementia unit investigates temperature and Behavioural and Psychological Symptoms of Dementia (BPSD). We sought to control for diurnality. Sundown Syndrome (SS) is emergence or worsening of BPSD in the late afternoon or early evening. The literature affords debate. Our methods of controlling for time as a confounder for temperature generated contributions which we offer here.

**Methods:**

Data were collected from two Older People's Organic wards within the Cumbria, Northumberland Tyne and Wear NHS Foundation Trust. Collection used the Trust's “Talk First” data system. That is an established, verified record, including “aggression” (non-contact) or “violence” (contact). Data from 16 months, September 2019 to January 2021 were analysed.

Patients had moderate or severe dementia. Wards care for a maximum of 14 patients and serve either men or women. Data for the communal corridor and day room of each ward were analysed. This gave four site

We used two methods. The first was basic, the overall histogram of incidents through the day.

The second analysis counts “incident signals” from each time or temperature. Each actual occurring combination of temperature and time is assigned a “cell”. The background rate of all incidents per all cells is known. Any incident in any rare cell has low binomial probability. Low probabilities mean high “signal”. The square of sums of signals across each hour provides each hour's “incident signal”.

**Results:**

Median ages were 79 (women) and 82 (men). There were 99 incidents.

The histogram has two peaks, around lunchtime and evening. Late afternoon is relatively safe. Thermal incident signals are summarised as moderately coherent. Diurnal incident signals controlling for temperature did not show any coherent trend.

**Conclusion:**

We proffer approaches for controlling for temperature and time of day. The project has limits. We have a small sample. We have not compared sunset times; but that is not relevant to the mid-day peak. We present secondary data from an evaluation aimed at temperature. More favourably this is an a priori sample, shows the same thing is two ways, and adds to debate on an important and critiqued construct. Though SS uses “sun” as a shorthand, any effect will be mediated bio-psychosocially via light, social interaction, heat, circadian rhythms, etc. Our data support social interaction more than time of day. This may add to or challenge SS as a construct.

